# Profiling Healthcare Professionals' Digital Health Competence and Associated Factors: A Cross‐Sectional Study

**DOI:** 10.1111/jan.70435

**Published:** 2025-12-07

**Authors:** Dania Comparcini, Valentina Simonetti, Melania Totaro, Marco Tomietto, Barbara Forastefano, Erika Jarva, Francesco Pastore, Flavia Minoia, Beatrice Gullo, Teresa Rea, Assunta Guillari, Kristina Mikkonen, Giancarlo Cicolini

**Affiliations:** ^1^ Interdisciplinary Department of Medicine ‘Aldo Moro’ University of Bari Bari Italy; ^2^ Center of Innovation in Nursing Research (CINR)—Center for Advanced Studies and Technology (CAST) ‘G. d'Annunzio’ University of Chieti‐Pescara Chieti Italy; ^3^ Department of Innovative Technologies in Medicine & Dentistry ‘G.d'Annunzio’ University of Chieti‐Pescara Chieti Italy; ^4^ Department of Biomedicine and Prevention ‘Tor Vergata’ University Rome Italy; ^5^ School of Healthcare and Nursing Sciences, Faculty of Health and Wellbeing Northumbria University Newcastle upon Tyne UK; ^6^ Research Unit of Health Sciences and Technology University of Oulu Oulu Finland; ^7^ Department of Precision and Regenerative Medicine and Jonian Area ‐ (DiMePRe‐J), Nephrology, Dialysis and Transplantation Unit University of Bari Aldo Moro Bari Italy; ^8^ Department of Surgical Sciences IRCCS «S.de Bellis » Castellana Grotte Bari Italy; ^9^ Department of Public Health University of Naples ‘Federico II’ Naples Italy; ^10^ Department of Translational Medical Sciences, Clinical Research Center DEMeTra University of Naples ‘Federico II’ Naples Italy; ^11^ Medical Research Center Oulu, University Hospital University of Oulu Oulu Finland

**Keywords:** cluster analysis, digital health competence, healthcare professionals, nursing personnel, organisational support, professional development

## Abstract

**Aim:**

To assess healthcare professionals' digital health competence and its associated factors.

**Design:**

Cross‐sectional study.

**Methods:**

The study was conducted from October 2023 to April 2024 among healthcare professionals in Italy, using convenience and snowball sampling. The questionnaire included four sections assessing: (i) socio‐demographic and work‐related characteristics; (ii) use of digital solutions as part of work and in free time, and communication channels to counsel clients in work; and DigiHealthCom and DigiComInf instruments including measurements of (iii) digital health competence and (iv) managerial, organisational and collegiality factors. K‐means cluster analysis was employed to identify clusters of digital health competence; descriptive statistics to summarise characteristics and ANOVA and Chi‐square tests to assess cluster differences.

**Results:**

Among 301 healthcare professionals, the majority were nurses (*n* = 287, 95.3%). Three clusters were identified: cluster 1 showing the lowest, cluster 2 moderate and cluster 3 the highest digital health competence. Most participants (*n* = 193, 64.1%) belonged to cluster 3. Despite their proficiency, clusters 2 and 3 scored significantly lower on ethical competence. Least digitally competent professionals had significantly higher work experience, while the most competent reported stronger support from management, organisation, and colleagues. Communication channels for counselling clients and digital device use, both at work and during free time, were predominantly traditional technologies.

**Conclusion:**

Educational programmes and organisational policies prioritising digital health competence development are needed to advance digital transition and equity in the healthcare workforce.

**Implications for the Profession:**

Greater emphasis should be placed on the ethical aspects, with interventions tailored to healthcare professionals' digital health competence. Training and policies involving managers and colleagues, such as mentoring and distributed leadership, could help bridge the digital divide. Alongside traditional devices, the adoption of advanced technologies should be promoted.

**Reporting Method:**

This study adheres to the STROBE checklist.

**Patient or Public Contribution:**

None.

## What Does This Paper Contribute to the Wider Global Clinical Community?

- This paper expands the understanding of patterns of digital health competence among healthcare professionals and its associated factors, drawing attention to the widespread underutilisation of advanced digital technologies among professionals.
- The findings provide valuable insights to inform strategies at the micro, meso and macro levels, helping to shape targeted actions that consider individual, organisational and systemic factors.

## 1 Introduction

The healthcare digital transformation is reshaping both service provision and patient care delivery, highlighting the urge for a digitally competent workforce. The integration of advanced digital tools and health information technologies into clinical practice enhances access to care, optimises healthcare operations and reduces costs, thereby contributing to greater sustainability of healthcare systems (Bailony 2024; Widjaja et al. 2024). Nevertheless, fully realising these benefits depends on healthcare professionals' ability to effectively engage with and utilise digital technologies (Kulju et al. 2024).

The existing literature on digital health competence among healthcare professionals can be misleading, as ‘competence’ and ‘literacy’ are often used synonymously or loosely, despite denoting distinct concepts (Longhini et al. 2022; Mainz et al. 2024). Digital health literacy refers to the ability to seek, find, understand and critically evaluate health information from electronic sources or digital contexts, and to apply the knowledge obtained to address or solve a health problem (Yang et al. 2022). Digital health competence extends beyond technical skills and information understanding to encompass psychological and emotional aspects, ethical management of sensitive content, communication and networking abilities and strategic leadership to support technology adoption within the workplace (Longhini et al. 2024; Mainz et al. 2024). It is shaped by personal attitudes towards digital health solutions, professional experience, consistent technology use and organisational factors such as collegial and managerial support (Konttila et al. 2019; Jarva, Mikkonen, et al. 2022). Additionally, sociodemographic and contextual variables, such as age, seniority, educational attainment and employment setting, contribute to the observed variability in digital health competence among healthcare professionals (Erfani et al. 2025; Jarva et al. 2024).

Younger healthcare professionals tend to feel more comfortable with digital solutions and exhibit a more positive attitude towards digitalization compared to their older colleagues (Jarva et al. 2024). Likewise, those with higher educational attainment show greater digital health competence (Erfani et al. 2025; Longhini et al. 2024). Nevertheless, accounting for individual variability, the successful scale‐up of digital healthcare depends not only on agents at the micro level but also on coordinated efforts at the meso and macro levels, where barriers often outweigh enablers (Schlieter et al. 2022).

Meanwhile, the rapid evolution of digital technologies risks outpacing the training and preparedness of the current healthcare workforce, thereby contributing to a growing digital skills gap (Morris et al. 2023). To address these issues, healthcare professionals' education curricula should be flexible enough to keep up with emerging technologies and promote digital health competences through the development of data management abilities and ethical decision‐making (Alhur 2023). However, improvements in education alone are not enough. Organisational enablers, such as a robust information technology infrastructure, continuous professional development pathways and a leadership culture that embraces technology‐driven innovation, can also contribute to advancing digital transformation within the healthcare workforce (Alotaibi et al. 2025; Brommeyer et al. 2024).

In this context, the digital health competence framework emphasises the relevance of integrating digital technologies into healthcare practice, highlighting not only information and communication technologies, their usability, and ethical use, but also the educational and organisational factors that influence the development of health competence within healthcare settings (Jarva et al. 2023). At the macro level, the European Union (EU) has undertaken several policies in support of this transition. The *Digital Competence Framework* strategy aimed to equip Europe with digitally empowered citizens and a digitally skilled workforce (European Commission 2021). The framework delineates the digital competence areas as information and data literacy, communication and collaboration, digital content creation, safety and problem solving. Building upon this, the *2030 Digital Compass* set an ambitious goal that a minimum of 80% of Europeans should have at least basic digital skills by 2030 (European Parliament and Council of the European Union 2022). However, despite such global efforts, noteworthy challenges in digital health competence persist. Among these, the equitable digitalisation of the health and care sector across Europe is hindered by a lack of digital skills among professionals, patients, carers and families (Healthy Europe 2023). This issue is reflected in national performance data. According to the *European Commission's Digital Economy and Society Index* (DESI) 2024, based on 2023 data, Italy ranked below the EU average, with more than half of the general adult population lacking at least basic digital skills (European Commission 2025).

Considering these challenges, it is worthwhile to evaluate healthcare professionals' digital health competence, which encompasses not only operational proficiency but also attitudes, evaluation, counselling and ethical competence, as well as the associated managerial, organisational and collegiality factors.

Traditional self‐report measures often focus narrowly on technical skills, overlooking critical areas such as organisational adaptability and critical thinking (Mainz et al. 2024). Rather, healthcare professionals' digital health competence requires a multidimensional assessment encompassing associated all‐level factors, in order to enable targeted training and effectively meet diverse needs (Ylönen et al. 2025). To date, although this topic has been addressed in a few studies (Erfani et al. 2025; Jarva et al. 2024) and reviews (Alotaibi et al. 2025; Longhini et al. 2024; Mainz et al. 2024), the influence of these factors can vary widely across geographical contexts and healthcare institutions (Gao et al. 2025), warranting further investigation. Furthermore, given the patterns observed in the general population, it is particularly valuable to explore digital health competence and its associated factors among Italian healthcare professionals.

## 2 The Study

### 2.1 Aim

This study aimed to assess healthcare professionals' digital health competence and its associated factors to identify distinct competence profiles using a clustering approach.

## 3 Methods

### 3.1 Design

A cross‐sectional study was carried out among healthcare professionals in Italy between October 2023 and April 2024. The reporting of this research adhered to the *Strengthening the Reporting of Observational Studies in Epidemiology* (STROBE) checklist (von Elm et al. 2008).

### 3.2 Study Participants and Sampling

Healthcare professionals were recruited using a convenience sampling strategy integrated with a snowball sampling approach. They were invited to participate through formal and informal networks, including social media platforms, academic networks and professional associations. All those who were currently practicing as healthcare professionals and provided informed consent were included.

### 3.3 Data Collection Procedures

Data were collected using an online survey implemented in the LimeSurvey platform. To prevent inappropriate access, a CAPTCHA system was adopted requiring participants to solve a basic addition problem to verify that the user was human and not an Internet bot. Additionally, a cookie‐based tracking system was deployed to prevent duplicate or multiple submissions from the same user's device. The first screen of the survey provided an information sheet explaining the study objectives, data anonymisation procedures, and the right to withdraw at any time. Participants could access the survey only after giving their informed consent. To enhance participation, weekly reminders were distributed via social media platforms and both formal and informal networks of healthcare professionals.

### 3.4 Questionnaire Description

The questionnaire used in the online survey was composed of the following four sections.

- *Socio‐demographic and work‐related characteristics*, including age, gender, educational level, professional role, type of work and years of work experience.
- *Use of digital solutions as part of work and in free time, and communication channels to counsel clients in work*, recording the self‐reported frequency on a 5‐point Likert scale (1 = ‘daily’, 2 = ‘weekly’, 3 = ‘monthly’, 4 = ‘occasionally’, 5 = ‘never’).
- *Digital health competence*, assessed through the DigiHealthCom instrument developed by Jarva et al. (2023), comprises 42 items across the following five factors: (1) *Human‐centred remote counselling competence* (*Counselling*), which refers to the ability to provide effective support and guidance in remote counselling contexts; (2) *Digital solutions as part of work* (*Attitude*), measuring attitude towards digital solutions at work and the extent of digital technology integration in professional activities; (3) *Information and communication technology competence* (*ICT*), which assesses proficiency in utilising ICT tools and software; (4) *Competence in utilising and evaluating digital solutions* (*Evaluation*), reflecting the ability to critically assess and effectively implement digital innovations in professional practice; (5) *Ethical competence related to digital solutions* (*Ethics*), which evaluates the ability to manage confidential information securely and ethically in digital environments.
- *Educational and organisational factors associated with digital health competence*, measured using the DigiComInf instrument (Jarva et al. 2023), consist of 15 items distributed across three factors: (1) *Support from management* (*Management*), determining the extent of organisational support for adopting digital technologies; (2) *Organisational practices as part of digital competence development* (*Organisation*), which evaluates institutional efforts to facilitate professional development in digital health competence; (3) *Colleagues' adoption and influence* (*Collegiality*), assessing how colleagues' openness, behaviours and digital health competence support the adoption of digital tools within the team.

All items of the DigiHealthCom and DigiComInf instruments were rated using a 4‐point Likert scale, from 1 = ‘completely disagree’ to 4 = ‘completely agree’.

### 3.5 Scales' Validity and Reliability

The original English versions of the DigiHealthCom and DigiComInf instruments were translated into Italian using the forward‐backward translation process described by Maneesriwongul and Dixon (2004) to ensure content validity. In particular, the instruments were first translated from English into Italian by a researcher fluent in both languages. An independent translator, blinded to the original versions, then performed a back‐translation into English. Finally, the original and back‐translated versions were compared by the authors of the original scales to ensure content equivalence between the two versions.

To provide linguistic and cultural adaptation for the national context, a panel of experts in research and clinical practice, not previously involved in the translation process, independently evaluated the scales. The panel assessed the relevance and clarity of each item, and the Content Validity Index (CVI) was calculated. Specifically, a total of six experts rated both scales for relevance and clarity on a 4‐point Likert scale (1 = ‘not relevant’/‘not clear’; 2 = ‘somewhat relevant’/‘somewhat clear’; 3 = ‘quite relevant’/‘quite clear’; 4 = ‘highly relevant’/‘highly clear’) where scores equal to 3 and 4 were considered as ‘relevant’ and ‘clear’ for the CVI calculation. Following the current standards (Polit and Beck 2006; Yusoff 2019), a cut‐off of 0.83 was applied at the scale level (S‐CVI) to determine adequate content validity. In this study, the relevance and clarity of the DigiHealthCom scale were 0.92 and 0.93 respectively, while the DigiComInf scale scored 0.79 for relevance and 0.84 for clarity. No adaptations were needed following the content validity process.

To evaluate instrument reliability, Cronbach's alpha was calculated. According to DeVellis (2016), reliability values > 0.90 are considered excellent, > 0.70 and < 0.90 good, > 0.60 and < 0.70 acceptable, and < 0.60 unacceptable. In this study, the DigiHealthCom and DigiComInf instruments showed overall Cronbach's alphas of 0.94 and 0.96, respectively, with factor values ranging from 0.80 to 0.96 for DigiHealthCom and between 0.94 and 0.95 for DigiComInf.

### 3.6 Data Analysis

K‐means cluster analysis was employed to identify clusters of digital health competence based on the DigiHealthCom overall mean score. K‐means clustering is an unsupervised machine learning technique that groups data into clusters according to their similarity (Ikotun et al. 2023). This clustering approach is useful for uncovering latent patterns within the sample distribution and describing their characteristics (Wagstaff et al. 2001). As it is an unsupervised method, K‐means clustering does not require predefined labels; instead, the algorithm autonomously detects underlying structures or patterns within the data. Data standardisation was not required, as all DigiHealthCom items were measured on a 4‐point Likert scale (Ikotun et al. 2023). The optimal number of clusters was determined through a two‐step cluster analysis, adopting silhouette measures of cohesion and separation, and applying a Euclidean distance metric. Based on the silhouette measure of cohesion and separation, the analysis confirmed that a three‐cluster solution was optimal for the dataset.

Descriptive statistics were utilised to summarise the characteristics of the identified clusters. No missing data were observed in the dataset. To categorise participants by generation, the classification criteria outlined by Schmitt and Lancaster (2019) were applied. In detail, individuals born between 1946 and 1964 were classified as Baby Boomers, those born between 1965 and 1980 as Generation X, those born between 1981 and 1996 as Generation Y, and those born after 1997 as Generation Z. One‐way ANOVA and chi‐squared tests were performed to evaluate the statistical significance of differences among clusters, with a *p*‐value < 0.05 considered statistically significant.

### 3.7 Sample Size Estimation

Given that K‐means clustering, employed in data analysis, is an unsupervised machine learning method, it was not possible to pre‐determine the required sample size for a specific number of clusters. Consequently, the sample size estimation was performed by considering a range of potential cluster solutions (three to five clusters). Based on an alpha level of 0.05, a statistical power of 0.95 and an effect size of 0.25, a sample size of 305 participants was recommended for five clusters, while 252 participants were estimated as sufficient for three clusters under the same statistical parameters.

### 3.8 Ethical Considerations

The study received approval from the Local Ethics Committee of the Polyclinic of Bari, Italy (protocol number: 0003531, approval date: 13/01/2023). All data collection and analysis procedures adhered to the European data protection regulations (European Parliament and Council of the European Union 2018). Participation in the survey was entirely voluntary, confidential and anonymous. Informed consent was obtained from each participant before they proceeded to the survey. The survey platform used a strong, password‐protected login system and all data was securely stored in a protected folder accessible only to the principal investigator.

## 4 Results

### 4.1 Characteristics of the Sample

A total of 301 healthcare professionals took part in the study, the majority of whom were nurses (*n* = 287, 95.3%), with the remaining participants consisting of allied healthcare professionals. The sample had a mean age of 39.4 years (SD = 11.4) and a mean work experience of 13.6 years (SD = 11.0). The majority held a Bachelor's degree (*n* = 240, 79.7%), while 18.3% (*n* = 55) had a Master's and 2.0% (*n* = 6) a PhD. Most of the participants were full‐time employees (*n* = 294, 97.7%). Regarding generational distribution, 3.0% of the sample belonged to the Baby Boomer generation (*n* = 9), 33.2% to Generation X (*n* = 100), 49.5% to Generation Y (*n* = 149) and 14.3% to Generation Z (*n* = 43).

### 4.2 Cluster Analysis

The two‐step cluster analysis identified three clusters of digital health competences as an optimal solution. Significant differences among the three clusters across all examined digital competences and associated factors were confirmed (*p* < 0.001).

#### 4.2.1 Cluster 1: Lowest Digital Health Competence and Engagement

Cluster 1 accounted for 8.6% of all respondents (*n* = 26) and showed the lowest mean scores in both overall DigiHealthCom (M = 1.9, SD = 0.3) and DigiComInf (M = 1.8, SD = 0.7), as well as across all factors, significantly lower than the scores observed in the other clusters (*p* < 0.001) (Table 1).

**TABLE 1:** Digital health competence and associated factors by cluster: ANOVA Results.

Scale/Factors	Cluster_1 (*n* = 26), mean (SD)	Cluster_2 (*n* = 82), mean (SD)	Cluster_3 (*n* = 193), mean (SD)	*F*	*p*
DigiHealthCom scale	1.9 (0.3)	2.8 (0.2)	3.2 (0.1)	690.023	**< 0.001**
Factor 1: Counselling	1.8 (0.5)	2.7 (0.4)	3.4 (0.4)	200.635	**< 0.001**
Factor 2: Attitude	2.0 (0.6)	3.2 (0.4)	3.7 (0.3)	221.352	**< 0.001**
Factor 3: ICT	2.3 (1.0)	3.4 (0.5)	3.7 (0.3)	105.633	**< 0.001**
Factor 4: Evaluation	1.8 (0.4)	2.4 (0.4)	2.5 (0.4)	33.493	**< 0.001**
Factor 5: Ethics	1.9 (0.5)	2.3 (0.4)	2.3 (0.4)	12.586	**< 0.001**
DigiComInf scale	1.8 (0.7)	2.4 (0.7)	2.6 (0.8)	13.690	**< 0.001**
Factor 1: Management	1.9 (0.7)	2.4 (0.8)	2.6 (0.9)	8.794	**< 0.001**
Factor 2: Organisation	1.6 (0.6)	2.2 (0.8)	2.5 (0.9)	12.868	**< 0.001**
Factor 3: Collegiality	1.9 (0.9)	2.6 (0.7)	2.7 (0.8)	10.620	**< 0.001**

*Note:* Statistically significant *p*‐values in bold.

Participants in this cluster were slightly older (mean age 43.5 years, SD = 11.6) compared to clusters 2 and 3, although this difference was not statistically significant, and generations X and Y were equally represented (42.3% each) (Tables 2 and 3). Moreover, cluster 1 participants had significantly higher work experience (M = 18.9 years, SD = 12.4) than those in clusters 2 and 3 (*p* = 0.038) (Table 2).

**TABLE 2:** Sample characteristics, the use of digital solutions as part of work and in free time and communication channels to counsel clients in work, by cluster: ANOVA results.

Variable		Cluster_1 (*n* = 26), mean (SD)	Cluster_2 (*n* = 82), mean (SD)	Cluster_3 (*n* = 193), mean (SD)	*F*	*p*
Age (years)		43.5 (11.6)	39.1 (11.6)	39.0 (11.2)	1.807	0.166
Work experience (years)		18.9 (12.4)	13.4 (10.7)	13.0 (10.7)	3.299	**0.038**
Use of digital solutions as part of my work	Computer	1.8 (1.4)	1.6 (1.2)	1.3 (0.8)	5.119	**0.007**
	Smartphone	2.7 (1.9)	2.2 (1.7)	1.9 (1.5)	3.452	**0.033**
	Tablet	3.9 (1.6)	4.2 (1.4)	3.9 (1.6)	1.086	0.339
	Wearable	4.0 (1.7)	4.2 (1.5)	4.2 (1.5)	0.185	0.831
	Robotics	4.4 (1.4)	4.8 (0.8)	4.7 (0.9)	2.355	0.097
	Digital services	3.4 (1.8)	2.7 (1.7)	2.5 (1.7)	4.009	**0.019**
	Well‐being apps	4.0 (1.7)	3.6 (1.7)	3.6 (1.7)	0.933	0.394
	Workspaces (e.g., Google Drive)	3.1 (1.9)	3.2 (1.6)	2.7 (1.7)	3.709	**0.026**
	Remote (e.g., Teams, Zoom)	4.0 (1.5)	4.0 (1.2)	3.7 (1.4)	1.301	0.274
	Consoles	4.7 (1.0)	4.9 (0.5)	4.8 (0.8)	0.646	0.525
Use of digital solutions in my free time	Computer	1.6 (1.2)	1.9 (1.2)	1.7 (1.1)	1.569	0.210
	Smartphone	1.2 (0.5)	1.1 (0.5)	1.1 (0.5)	0.312	0.733
	Tablet	3.9 (1.5)	3.7 (1.5)	3.5 (1.6)	1.086	0.339
	Wearable	3.7 (1.9)	3.6 (1.8)	3.5 (1.7)	0.047	0.954
	Robotics	4.3 (1.5)	4.7 (0.9)	4.7 (0.9)	2.186	0.114
	Digital services	3.0 (1.8)	3.0 (1.5)	2.8 (1.6)	0.590	0.555
	Well‐being apps	3.5 (1.8)	3.2 (1.5)	3.2 (1.6)	0.531	0.588
	Workspaces (e.g., Google Drive)	2.7 (1.8)	2.7 (1.5)	2.1 (1.4)	6.646	**0.002**
	Remote (e.g., Teams, Zoom)	3.7 (1.6)	3.6 (1.2)	3.2 (1.3)	2.623	0.074
	Consoles	4.5 (1.2)	4.5 (0.9)	4.5 (1.0)	0.036	0.965
In my work, I counsel clients…	Face to face	2.0 (1.6)	1.8 (1.4)	1.6 (1.2)	1.257	0.286
	Face‐to‐face via video link	4.2 (1.4)	4.7 (0.7)	4.5 (1.0)	4.232	**0.015**
	By telephone	3.0 (1.6)	3.3 (1.6)	2.8 (1.7)	2.686	0.070
	Via text (e.g., emails, SMS)	3.9 (1.6)	4.0 (1.4)	3.4 (1.7)	5.257	**0.006**
	Via chat	4.4 (1.3)	4.2 (1.3)	3.8 (1.5)	3.245	**0.040**

*Note:* Statistically significant *p*‐values in bold.

**TABLE 3:** Sociodemographic characteristics by cluster: chi‐squared test.

Variable	Category	Cluster_1 (*n* = 26), *n* (%)	Cluster_2 (*n* = 82), *n* (%)	Cluster_3 (*n* = 193), *n* (%)	Chi‐squared	df	*p*
Gender	Female	14 (53.8%)	54 (65.9%)	129 (66.8%)	3.847	4	0.427
	Male	11 (42.4%)	27 (32.9%)	63 (32.7%)			
	Prefer not to say	1 (3.8%)	1 (1.2%)	1 (0.5%)			
Education	Bachelor	22 (84.6%)	73 (89.0%)	145 (75.1%)	8.620	4	0.071
	Master	4 (15.4%)	7 (8.5%)	44 (22.8%)			
	PhD	0 (0.0%)	2 (2.4%)	4 (2.1%)			
Professional role	Nurse	22 (84.6%)	79 (96.3%)	186 (96.4%)	7.393	2	**0.025**
	Others	4 (15.4%)	3 (3.7%)	7 (3.6%)			
Type of work	Full‐time	26 (100.0%)	78 (95.1%)	190 (98.4%)	3.476	2	0.176
	Part‐time	0 (0.0%)	4 (4.9%)	3 (1.6%)			
Generations	BB	2 (7.7%)	2 (2.4%)	5 (2.6%)	4.068	6	0.667
	X	11 (42.3%)	26 (31.7%)	63 (32.6%)			
	Y	11 (42.3%)	41 (50.0%)	97 (50.3%)			
	Z	2 (7.7%)	13 (15.9%)	28 (14.5%)			

*Note:* Statistically significant *p*‐values in bold.

In terms of devices used at work, the usage of smartphones (*p* = 0.033) and digital services (*p* = 0.019) varied significantly across clusters, with smartphones being used almost monthly (M = 2.7, SD = 1.9) and digital services tending towards occasional use (M = 3.4, SD = 1.8) in cluster 1.

Face‐to‐face interaction in the same physical space was the most frequently used channel to counsel clients across all clusters, occurring slightly less than weekly on average, with no significant differences observed. However, cluster 1 reported significantly higher usage of face‐to‐face interaction via video link (M = 4.2, SD = 1.3) compared to clusters 2 and 3 (*p* = 0.015), although this still reflected an occasional‐to‐rare frequency of use (Table 2).

#### 4.2.2 Cluster 2: Moderate Digital Health Competence and Engagement

Accounting for 27.2% of respondents (*n* = 82), cluster 2 significantly differed from the others (*p* < 0.001), showing intermediate scores in DigiHealthCom (M = 2.8, SD = 0.2), DigiComInf (M = 2.4, SD = 0.7) and across all factors (Table 1). Reflecting a similar pattern to cluster 3, half of the participants in cluster 2 belonged to generation Y (Table 3).

Workspace usage also differed significantly across clusters both during work (*p* = 0.026) and free time (*p* = 0.002). Specifically, cluster 2 reported the lowest workspace usage at work, occurring just about monthly (M = 3.2, SD = 1.6), while during free time, usage was slightly more frequent, close to monthly (M = 2.7, SD = 1.5), similar to cluster 1 (M = 2.7, SD = 1.8). Finally, participants in cluster 2 used face‐to‐face interaction via video link to counsel clients significantly less than those in clusters 1 and 3 (*p* = 0.015), with a mean frequency approaching never (M = 4.7, SD = 0.7) (Table 2).

#### 4.2.3 Cluster 3: Highest Digital Health Competence and Engagement

The majority of participants (*n* = 193, 64.1%) were in cluster 3, which recorded significantly the highest average scores for both overall DigiHealthCom (M = 3.2, SD = 0.1) and DigiComInf (M = 2.6, SD = 0.8) as well as across all related factors (*p* < 0.001) (Table 1).

This cluster also had the lowest mean age (M = 39.0, SD = 11.2) and the highest proportion of female participants (66.8%). Half of its members belonged to generation Y (50.3%), and 22.8% had a Master's degree (22.8%). However, these differences were not statistically significant (Tables 2 and 3). A statistically significant difference between clusters was instead observed with respect to professional role (*p* = 0.025), with nurses being more highly represented in this cluster (96.4%). Work experience in cluster 3 was significantly lower than in the other clusters (M = 13.0 years, SD = 10.7, *p* = 0.038) (Table 2).

Exploring technology use, the computer emerged as the most frequently used digital solution as part of work across all clusters, with usage being significantly higher in cluster 3 (M = 1.3, SD = 0.8, *p* = 0.007). Additionally, when examining communication channels used to counsel clients at work, statistically significant differences were observed across clusters in the roughly monthly and occasional use of both via text (*p* = 0.006) and via chat communication (*p* = 0.040), with cluster 3 showing slightly higher usage rates (M = 3.4, SD = 1.7; M = 3.8, SD = 1.5, respectively) (Table 2).

## 5 Discussion

This study aimed to explore healthcare professionals' digital health competence and its associated factors, contributing new evidence to the existing literature on this topic within a different geographical context and healthcare system. Moreover, this study adopted a novel methodological approach to disclose possible hidden patterns and characteristics of nurses' and healthcare professionals' digital health competence. Through cluster analysis, three distinct profiles of digital health competence emerged. The results revealed some key elements that should be considered when promoting these skills, informing strategies at the micro, meso and macro levels.

### 5.1 Digital Health Competence Within Clusters

The identification of three clusters of low, moderate and high digital health competence reflects patterns observed in recent studies among healthcare professionals' (Erfani et al. 2025; Jarva et al. 2024; Ylönen et al. 2025). While it is encouraging that the majority of the sample belonged to cluster 3 (highest digital health competence), the differences between clusters suggest that tailored, multi‐tiered interventions are warranted. Notably, this study found that clusters 2 (moderate digital health competence) and 3, scored lowest on the ethical dimension, suggesting that although these professionals were relatively skilled, their understanding of ethical issues in the context of digital solutions remained limited. Comprehension of ethical considerations, such as privacy, freedom of choice, autonomy, fairness and data protection, cannot be overlooked when engaging with technologies (Mainz et al. 2024). Therefore, strong ethical and legal foundations are needed to enhance healthcare professionals' awareness in digital contexts (Galazzi et al. 2025; Kulju et al. 2024). In light of these findings, continuous professional development initiatives and educational programmes for healthcare professionals in cluster 1 should prioritise the development of basic digital health competences, whereas those for clusters 2 and 3 should focus on promoting the ethical use of digital technologies.

### 5.2 Clusters, Educational and Organisational Factors Associated With Digital Health Competence

Healthcare professionals in cluster 3, who demonstrated the highest digital proficiency, also reported stronger support from management, organisation and colleagues. Previous research confirms that healthcare professionals who perceive strong organisational backing are more likely to engage with and adopt digital health solutions (Erfani et al. 2025; Kulju et al. 2024). Drawing from this study's findings, adequate organisational support and encouragement should be provided to successfully help professionals in developing digital health competence (Kulju et al. 2024). Greater emphasis could further be placed on distributed leadership, in which middle managers and colleagues proactively contribute to the development of healthcare professionals' digital skills, without neglecting the increased job satisfaction that healthcare staff might experience compared to a hierarchical model (Leask and Macleod 2023).

### 5.3 Clusters, Socio‐Demographic and Work‐Related Characteristics

Cluster 1, with the lowest digital health competence, had higher age and work experience, although only the latter was statistically significant. This aligns with international studies indicating that younger healthcare professionals or those with fewer years in practice are more likely to be digitally competent (Erfani et al. 2025; Jarva et al. 2024). Senior professionals may face challenges adapting to digital tools due to limited prior exposure, insufficient training opportunities or resistance to change (Jarva et al. 2024). These findings corroborate the need for continuous digital education throughout all career stages to ensure they receive adequate support in embracing digital solutions. Studies have shown that intergenerational mentoring, where younger, digitally proficient employees aid more experienced colleagues, can be an effective strategy for closing the digital divide in healthcare (Hammarén et al. 2022; Kulju et al. 2024). Therefore, younger and more digitally competent professionals could benefit from advanced training in mentoring roles to support senior colleagues in developing digital health competences within their teams. Policy measures could also consider framing healthcare professionals' digital training as working time, with incentives and rewards to promote participation and commitment (Galazzi et al. 2025). By focusing on specific healthcare workers who may be disadvantaged, these efforts could promote equity (Longhini et al. 2024) and address the training gap that limits the application of emerging technologies in healthcare settings (Khan et al. 2025).

In addition, the chi‐squared analysis revealed no significant differences in education level among clusters. Nevertheless, cluster 3 (highest competence) included the largest proportion of participants with a Master's degree, followed by clusters 2 (moderate) and 1 (lowest). This trend is consistent with prior research indicating that healthcare professionals with higher qualifications exhibited greater digital health competence in their roles (Erfani et al. 2025). However, as observed by Jarva et al. (2024), this pattern did not reach statistical significance in this study, suggesting that formal education alone does not fully explain the differences in competence. Supporting this, a systematic review of digital education programmes for healthcare professionals found that blended learning approaches, combining traditional instruction with hands‐on digital training, significantly improve digital health competence (Kulju et al. 2024). In parallel, ensuring quality, equity and consistency in education requires macro‐level policies for systematic national coordination (Kaihlanen et al. 2024). Insights related to professional roles also emerged. While previous research (Jarva et al. 2024) suggested that nurses predominantly belonged to the lowest digital health competence cluster, this study found a statistically significant difference by professional role, with most nurses in the moderate or highest digital health competence clusters. Nevertheless, these results were not exempt from the influence of sociodemographic, educational and organisational factors.

### 5.4 Clusters and Use of Digital Solutions as Part of Work and in Free Time

Beyond the commonplace usage of traditional devices such as computers and smartphones, the adoption of other sophisticated technologies such as tablets, wearables and well‐being apps was limited. Consoles and robotics were the least utilised across all clusters, consistent with Jarva et al. (2024). Investigating how workers manage their digital time during leisure and work could offer valuable insights, as leisure activities are typically enjoyable and voluntarily chosen (Gellmers and Yan 2023). Moreover, since digital health competence is influenced by personal attitudes and experiences (Konttila et al. 2019), the results indicate limited prospects for transferring such behaviours from non‐professional contexts, where they also remain underdeveloped. The findings suggest that despite rapid advances in robotics, clinical decision support systems and artificial intelligence applications, these technologies have not yet reached broad uptake or feasibility within the healthcare sector (Jarva et al. 2024). Accordingly, healthcare professionals in cluster 1 (lowest competence) may require basic digital literacy training; those in cluster 2 (moderate competence) could benefit from slightly more sophisticated technology modules, while for those in cluster 3 (highest competence), efforts should focus on providing expert training to improve their skills in areas such as artificial intelligence, robotics and big data.

### 5.5 Clusters and Communication Channels to Counsel Clients in Work

Face‐to‐face interaction in the same physical space was the most frequently used channel for counselling clients across all clusters, underscoring the perceived irreplaceability of direct human contact in therapeutic relationships. Conversely, face‐to‐face interaction via video link was the least used channel in cluster 2 (moderate competence) and cluster 3 (highest competence), with the latter reporting the lowest usage, possibly indicating a transitional stage: not fully confident in digital tools, yet not entirely resistant either. According to Jarva, Oikarinen, et al. (2022), healthcare professionals acknowledged that while digital health services are beneficial for certain therapies and follow‐up visits, some procedures and assessments, such as rehabilitation evaluations, still require in‐person care. Among other communication channels, emails and SMS were deemed suitable depending on the care needed (Mazouri‐Karker et al. 2023), and cluster 3, the most digitally competent, used them more frequently. Still, the second most used tool across all clusters was the telephone. This aligns with previous findings showing that patients preferred phone consultations over video due to their simplicity of use and inexperience with video tools (Snoswell et al. 2024). Furthermore, they also perceive phone calls as safer than video consultations, as they do not permit visual examination or non‐verbal communication (Mazouri‐Karker et al. 2023). Although the video‐based channel offers greater diagnostic accuracy and fewer therapeutic errors (Mazouri‐Karker et al. 2023), the frequency results observed in this study may reflect patient preferences and digital familiarity. In this vein, a hybrid model tailored to patients' digital abilities and willingness seems to be the most suitable approach (Jarva, Oikarinen, et al. 2022).

### 5.6 Limitations and Strengths of the Work, and Recommendations for Further Research

The cross‐sectional design allowed the identification of potential factors associated with digital health competence, but it restricted the ability to infer causal or temporal relationships. A longitudinal design could offer a deeper understanding of how digital health competence changes over time among healthcare professionals. Notably, this study benefited from cluster analysis, which allowed the identification of three distinct digital health competence profiles among Italian healthcare professionals, uncovering latent patterns and segmenting the sample into meaningful groups without predefined categories. The convenience and snowball sampling, along with the online data collection on a voluntary basis, may have introduced selection bias by unintentionally favouring respondents with certain digital health competence, limiting generalisability. Data were collected via a self‐assessment questionnaire, potentially influenced by response bias and social desirability. A strength of this study is the use of two validated tools to assess digital health competences of healthcare professionals at both individual and organisational levels. The strong representation of nurses in the sample should not be viewed entirely as a limitation, given that they constitute the largest professional group in the healthcare sector (World Health Organization 2020). Nonetheless, this may restrict the transferability of the findings to other healthcare professional groups. Moreover, the predominance of participants belonged to generations X and Y, which may have affected the identification of significant differences by generational category. However, this distribution is to be expected, as both generation Z and the Baby Boomer cohort are naturally underrepresented in the current healthcare workforce, respectively due to age at workforce entry following graduation and retirement. Forthcoming studies should ensure wider, equitable representation of professional roles and generational categories to improve accuracy and generalisability, while minimising potential biases related to sample composition.

## 6 Conclusion

This study deepened the understanding of digital health competence and its patterns among healthcare professionals. Three digital health competence clusters were identified: cluster 1 (lowest), cluster 2 (moderate) and cluster 3 (highest), with the majority in cluster 3. However, even the most skilled scored lowest on the ethical factor. Those with the lowest competence had the most work experience, while those with the highest competence reported stronger support from management, organisation and colleagues. Digital devices use was predominantly limited to traditional ones, both at work and during free time. Face‐to‐face interaction was the most widely used counselling channel across clusters, whereas video link was least used by professionals with high and moderate competence. Targeted educational programmes and organisational policies prioritising digital health competence development are needed. Furthermore, interventional studies could explore the effectiveness of different strategies to support the development of digital health competences among healthcare professionals.

## Author Contributions

**Dania Comparcini:** conceptualization, methodology, writing – original draft preparation, supervision, writing – reviewing and editing. **Valentina Simonetti:** conceptualization, methodology, writing – original draft preparation, supervision, writing – reviewing and editing. **Melania Totaro:** writing – original draft preparation, visualisation, writing – reviewing and editing. **Marco Tomietto:** conceptualization, methodology, software, formal analysis, data curation, writing – original draft preparation, supervision, validation, writing – reviewing and editing. **Barbara Forastefano:** writing – original draft preparation, visualisation, writing – reviewing and editing. **Erika Jarva:** conceptualization, writing – reviewing and editing. **Francesco Pastore:** visualisation, writing – reviewing and editing. **Flavia Minoia:** visualisation, writing – reviewing and editing. **Beatrice Gullo:** visualisation, writing – reviewing and editing. **Teresa Rea:** investigation. **Assunta Guillari:** investigation. **Kristina Mikkonen:** conceptualization, methodology, writing – original draft preparation, supervision, writing – reviewing and editing. **Giancarlo Cicolini:** conceptualization, methodology, writing – original draft preparation, supervision, writing – reviewing and editing.

## Funding

The authors have nothing to report.

## Disclosure

Statistics: There is an expert statistician on the author team: Professor Marco Tomietto, Department of Nursing, Midwifery, and Health, Faculty of Health and Life Sciences, Northumbria University, Newcastle upon Tyne, United Kingdom.

## Conflicts of Interest

The authors declare no conflicts of interest.

## Data Availability

The data that support the findings of this study are available from the corresponding author upon reasonable request.
